# Uncovering the genomic heterogeneity of multifocal breast cancer

**DOI:** 10.1002/path.4540

**Published:** 2015-05-07

**Authors:** Christine Desmedt, Debora Fumagalli, Elisabetta Pietri, Gabriele Zoppoli, David Brown, Serena Nik‐Zainal, Gunes Gundem, Françoise Rothé, Samira Majjaj, Anna Garuti, Enrico Carminati, Sherene Loi, Thomas Van Brussel, Bram Boeckx, Marion Maetens, Laura Mudie, Delphine Vincent, Naima Kheddoumi, Luigi Serra, Ilaria Massa, Alberto Ballestrero, Dino Amadori, Roberto Salgado, Alexandre de Wind, Diether Lambrechts, Martine Piccart, Denis Larsimont, Peter J Campbell, Christos Sotiriou

**Affiliations:** ^1^ Breast Cancer Translational Research LaboratoryUniversité Libre de Bruxelles, Institut Jules BordetBoulevard de Waterloo 121BrusselsBelgium; ^2^ Department of Medical OncologyIstituto Scientifico Romagnolo per lo Studio e la Cura dei Tumouri (IRST) – IRCCSMeldolaItaly; ^3^ Department of Internal MedicineUniversity of Genoa and IRCCS Azienda Ospedaliera Universitaria San Martino – ISTGenoaItaly; ^4^ Cancer Genome ProjectWellcome Trust Sanger InstituteWellcome Trust Genome CampusHinxtonCambridgeshireUK; ^5^ Translational Breast Cancer Genomics LabDivision of Research, Peter MacCallum Cancer CentreEast MelbourneVictoriaAustralia; ^6^ VIB Vesalius Research Center, KU LeuvenCampus Gasthuisberg, Herestraat 49, Bus 912LeuvenBelgium; ^7^ Pathology Unit‘G.B. Morgagni–L. Pierantoni’ HospitalForlìItaly; ^8^ Breast International Group Headquarters (BIG‐aisbl)BrusselsBelgium; ^9^ Pathology Department, Jules Bordet InstituteBoulevard de Waterloo 121BrusselsBelgium; ^10^ Department of Medical OncologyJules Bordet InstituteBoulevard de Waterloo 121BrusselsBelgium; ^11^ Department of HaematologyUniversity of CambridgeCambridgeUK; ^12^ Department of HaematologyAddenbrooke's HospitalCambridgeUK

**Keywords:** breast cancer, multifocal, multicentric, targeted sequencing, genomic heterogeneity, oncogenic mutations

## Abstract

Multifocal breast cancer (MFBC), defined as multiple synchronous unilateral lesions of invasive breast cancer, is relatively frequent and has been associated with more aggressive features than unifocal cancer. Here, we aimed to investigate the genomic heterogeneity between MFBC lesions sharing similar histopathological parameters. Characterization of different lesions from 36 patients with ductal MFBC involved the identification of non‐silent coding mutations in 360 protein‐coding genes (171 tumour and 36 matched normal samples). We selected only patients with lesions presenting the same grade, ER, and HER2 status. Mutations were classified as ‘oncogenic’ in the case of recurrent substitutions reported in COSMIC or truncating mutations affecting tumour suppressor genes. All mutations identified in a given patient were further interrogated in all samples from that patient through deep resequencing using an orthogonal platform. Whole‐genome rearrangement screen was further conducted in 8/36 patients. Twenty‐four patients (67%) had substitutions/indels shared by all their lesions, of which 11 carried the same mutations in all lesions, and 13 had lesions with both common and private mutations. Three‐quarters of those 24 patients shared oncogenic variants. The remaining 12 patients (33%) did not share any substitution/indels, with inter‐lesion heterogeneity observed for oncogenic mutation(s) in genes such as PIK3CA, TP53, GATA3, and PTEN. Genomically heterogeneous lesions tended to be further apart in the mammary gland than homogeneous lesions. Genome‐wide analyses of a limited number of patients identified a common somatic background in all studied MFBCs, including those with no mutation in common between the lesions. To conclude, as the number of molecular targeted therapies increases and trials driven by genomic screening are ongoing, our findings highlight the presence of genomic inter‐lesion heterogeneity in one‐third, despite similar pathological features. This implies that deeper molecular characterization of all MFBC lesions is warranted for the adequate management of those cancers. © 2015 The Authors. Pathological Society of Great Britain and Ireland.

## Introduction

Multifocal or multicentric breast cancers are traditionally defined by the presence of two or more neoplastic lesions within a single breast quadrant or within different quadrants of the same breast, respectively 1. Since these definitions do not follow any internal anatomical boundary, here we use the term multifocal breast cancer (MFBC) to denote any ipsilateral, synchronous tumours presenting with separate invasive lesions. The two largest studies so far investigating the incidence of MFBC reported multifocality in 21% and 24% of patients, respectively 2, 3. No differences between multifocal and unifocal breast cancers have been reported in the distribution of molecular subtypes defined by oestrogen receptor (ER), progesterone receptor (PR), HER2, and the basal‐like CK5/6, CK14, and EGFR markers 4, suggesting that multifocality is not enriched in a certain molecular subtype. MFBC has instead been associated with a potentially worse clinical outcome and with increased axillary nodal involvement than unifocal tumours in most but not all publications 3, 5, 6, 7, 8, 9, 10, 11, 12, 13.

Based on the rationale that lesions with similar histology and grade are expected to be biologically similar, the College of American Pathologists recommends further characterizing all MFBC foci only when histology and grade differ from one another 14. However, recent studies question this recommendation because of well‐documented intra‐patient, inter‐lesion discordances in ER and HER2 expression (up to 4.4% and 10% discrepancy, respectively) 15, 16, 17. We therefore aimed to go a step further and assessed the potential genomic differences between ductal MFBC lesions with concordant histological grade, ER, and HER2 status, which represent the majority of MFBCs. To this end, we sequenced multiple lesions from a cohort of 36 patients with MFBC using a panel of 360 cancer‐related genes and validated this analysis by ultra‐deep resequencing using an orthogonal sequencing platform. For a subset of patients, we further characterized genome‐wide structural variations to interrogate the phylogenetic relationship between the various lesions.

## Materials and methods

### Patient selection

Patients were retrospectively selected on the basis of the following criteria: (1) documented MFBC, defined in the pathology report as the presence of multiple synchronous ipsilateral invasive lesions in the surgical specimen separated by benign breast tissue; (2) ductal histology; (3) tumour cellularity greater than 40%; (4) availability of two or more MFBC lesions, from which a minimum amount of 700 ng of double‐stranded DNA (dsDNA) could be extracted; (5) similar histological grade, ER, and HER2 status following central pathology review of the different lesions of the same patient; (6) no neo‐adjuvant treatment; and (7) availability of germline DNA, derived from whole blood or a tumour‐free axillary lymph node.

All 36 patients were diagnosed and operated on at Institut Jules Bordet or Ospedale Morgagni–Pierantoni (Forli, Italy) between 2000 and 2013. Eight of the 36 patients were selected for a whole‐genome rearrangement screen based on the availability of a frozen sample of at least two invasive lesions, from which a minimum of 3 µg of dsDNA could be extracted (Supplementary Tables 1 and 2), the remaining tissue samples used for this project all being formalin‐fixed, paraffin‐embedded (FFPE). The project was approved by the internal ethics committees of the two contributing hospitals (internal numbers and dates: 1748‐19/08/2010 and 2028‐11/10/2012) and conducted according to the principles of the Declaration of Helsinki.

### Targeted gene screen

The exonic regions of 360 cancer‐related genes (Supplementary Table 3) were enriched using in‐solution RNA baits (SureSelect, Agilent, UK) and sequenced on an Illumina HiSeq 2000 instrument at the Wellcome Trust Sanger Institute. Samples from eight patients were processed with an earlier version of the bait design, lacking 46 of those 360 genes (Supplementary Tables 2 and 3). Seven hundred nanograms to one microgram of DNA was fragmented to an average insert size of 145 bp (75–300) and subjected to Illumina DNA sequencing library preparation using the Bravo Automated Liquid Handling Platform. Individual samples were indexed using a unique DNA barcode via six cycles of PCR. Equimolar pools of 16 libraries were prepared and hybridized to custom RNA baits following the Agilent SureSelect protocol and sequenced on the Illumina HiSeq devices using the 75‐base pair paired‐end protocol. Somatic base substitutions and small insertions or deletions were identified by comparison with the matched normal sample using established bioinformatic algorithms 18, 19.

All mutations identified in at least one sample from a patient were further interrogated by deep resequencing using the Life Technologies Ion Torrent™ technology and confirmed to be present when the frequency of mutated reads was ≥ 5%, as in ref 20. Mutations with a frequency ≥ 1% but < 5% were defined as present only if there was at least one other sample in the same patient reporting the same mutation with a mutant allele fraction ≥ 5%. In brief, samples were analysed with heavily multiplexed primer pools, encompassing a total of 295 amplicons, designed with AmpliSeq^®^ Designer v 3.0.1 (Life Technologies Inc, Carlsbad, CA, USA). Targeted resequencing was performed by barcoding samples on 318™ chips and running the analyses on a PGM™ device (Life Technologies Inc), using the Torrent Suite pipeline and default parameters for FFPE material.

The sequencing data are available through the EBI's European Genome–phenome Archive under accession number EGAD00001001041.

### Identification of oncogenic mutations

Mutations were classified in one of the following categories according to the following definition, slightly modified from Papaemmanuil *et al* 21:

1. Oncogenic: non‐synonymous substitutions or in‐frame mutations in canonical oncogenes at recurrent hotspots; non‐synonymous substitutions recurrent in two or more confirmed samples in COSMIC; non‐synonymous substitutions recurrent in two or more samples in our own dataset; and nonsense, frameshifting insertions, and deletions in known tumour suppressors;
2. Putative oncogenic: previously unreported non‐synonymous substitutions in a known cancer gene within ±3 amino acids of a mutation recurrent in two or more samples in COSMIC; more than two non‐synonymous substitutions within three amino acids of each other (mutation clusters);
3. Possible oncogenic: non‐synonymous substitutions confirmed somatic in one sample in COSMIC; mutations close to mutation clusters in COSMIC;
4. Unknown significance: all remaining mutations.

For the sake of simplicity, all oncogenic, putative, and possible oncogenic mutations are referred to as ‘oncogenic’ here.

### Whole‐genome sequencing

Tumour genomic DNA from frozen multifocal lesions and matched germline DNA from eight patients were prepared for Illumina paired‐end sequencing according to the standard protocol (Illumina Inc, San Diego, CA, USA). Short reads were mapped back to the reference genome (GRCh37) and discordantly mapping reads were identified as pairs that did not match with the expected insert size, mapped in the wrong orientation, or mapped to different genomic regions, as previously described 22, 23. Putative rearrangements were validated and breakpoint annotated using capillary sequencing. Copy number alterations were assessed by QDNAseq (version 1.0.5) 24 by first binning the reads in non‐overlapping 100 kb windows. Bins in problematic regions were blacklisted. Read counts were corrected for GC content and mappability using a LOESS regression and median normalized. Raw log_2_ ratios were smoothed to remove outlier points using the median absolute deviation and segmented using the multitrack PCF algorithm as implemented in the R package copynumber (version 1.6.0) 25. Absolute estimates of copy numbers were obtained using ABSOLUTE (version 1.0.6) 26 in ‘total’ copy number mode and possible solutions of ploidy and cancer cell fraction were manually reviewed as explained on the software's webpage. Distance measures between lesions of a given patient were computed using a one‐step minimum event distance metric as implemented in the package MEDICC (version 10/2014) 27. We further compared the copy number status for the breast cancer‐specific focal copy‐number aberrations previously described in ref 28.

### Statistical analyses

Spearman's ρ was used to assess the correlation between allelic frequencies obtained with Illumina and Ion Torrent technologies. Wilcoxon's tests were used to test the significance of non‐zero shift for samples from two categories and Kruskal–Wallis tests were used to test whether samples for more than two categories had the same distribution. Fisher's exact tests were used for the analysis of contingency tables. All tests rejected the null hypothesis with a two‐tailed *p* value of 0.05.

Additional methods are reported in the Supplementary methods.

## Results

### Targeted sequencing of cancer genes in MFBC

To investigate the potential genomic heterogeneity between ductal MFBC lesions with centrally confirmed homogeneity in grade, ER, and HER2 status, we conducted targeted sequencing of cancer‐related genes in multifocal lesions from 36 patients (Table 1 and Supplementary Tables 1–3). Whenever lesion size allowed, we interrogated multiple geographically distinct samples per lesion, leading to a total of 171 investigated tumour samples. Sequencing was carried out at a median exonic coverage of 178×. Overall, 474 somatic mutations were identified, corresponding to 145 and 55 unique coding non‐silent somatic substitutions and indels, respectively, across all samples (Supplementary Table 4).

**Table 1:** Patient characteristics

	**Total No of patients**
Age, years	
<40	2
40–49	11
50–69	17
>70	6
Tumour size, cm	
1–2	17
2–5	18
>5	1
No of positive nodes	
None	16
1–3	16
4–9	1
>10	3
Tumour grade	
G1	8
G2	7
G3	21
Molecular subtype	
ER+/HER2−	26
ER−/HER2−	4
HER2+	6
DCIS	
Absent	6
Present	30
LVI	
Absent	21
Present	14
Unknown	1
Inter‐lesion distance*, cm	
<2	10
≥2	16
Unknown	10
No of lesions	
2	22
>2	14

DCIS = ductal carcinoma *in situ*; LVI = lymphovascular invasion.

* When there were more than two lesions, the largest inter‐lesion distance was taken into consideration for this table and for the downstream analyses.

To independently confirm the presence or absence of these mutations, we performed a second targeted sequencing experiment at greater sequencing depth (median coverage of 1344×) using an alternative platform (Supplementary Figure 1A). We could not confirm 35 mutations (7%), due to either design/technical failures (*n =* 19 mutations) or the lack of remaining DNA for four samples (*n =* 16 mutations). We validated 389 mutations among the remaining 439 (89%, referred to as ‘confirmed’ in Supplementary Table 4) and observed a strong correlation between the allelic frequencies reported by the two sequencing technologies (ρ = 0.81, Supplementary Figures 1B and 1C). The area under the ROC curve of Illumina and PGM sequencing, which can be considered as the probability to classify as positive a randomly chosen positive observation with the first method, had a value of 78.94% (95% CI = 71.25–86.62%). Fifty mutations could not be detected above the predefined cut‐off despite sufficient coverage at that specific genomic location. Of interest, this additional sequencing step also identified the presence of 52 additional mutations in samples where the mutation was previously undetected though present in other sample(s) from the same patient (referred to as ‘present’ in Supplementary Table 4).

We observed a median of three mutations (range 1–27) per patient. The number of mutations detected per patient correlated neither with the number of lesions nor with the number of samples sequenced (Supplementary Figures 2A and 2B). Of note, among the 141 validated unique mutations, 62 (44%) were identified as oncogenic (see the Materials and methods section) and thus susceptible to having contributed to the development of the cancer.

### Identification of inter‐lesion heterogeneity

**Figure 1:**
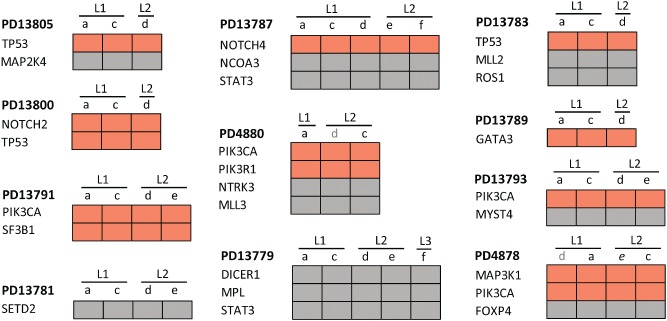
Distribution of non‐silent substitutions and indels in the ‘homogeneous’ MFBC group. Heat maps representing the non‐silent exonic substitutions and indels of the homogeneous multifocal lesions (L), which have all the mutations in common between all the lesions. Orange indicates the presence of an oncogenic mutation; grey the presence of a mutation of unknown significance; and white the absence of the mutation. When a sample name is grey, it means that it was not initially sequenced, due to a lack of sufficient DNA, but was included in the validation phase.

**Figure 2:**
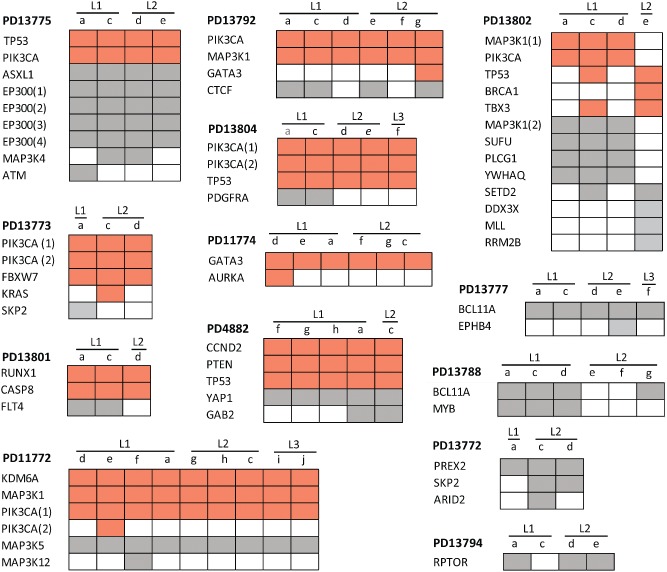
Distribution of non‐silent substitutions and indels in the ‘intermediate’ MFBC group. Heat maps representing the non‐silent exonic substitutions and indels of the intermediate multifocal lesions, presenting both common and private mutations. The colour code, abbreviations, and meaning of the grey colour for sample names are the same as in Figure 1. When numbers are present after the gene symbol, it means that different mutations were observed in the same gene.

**Figure 3:**
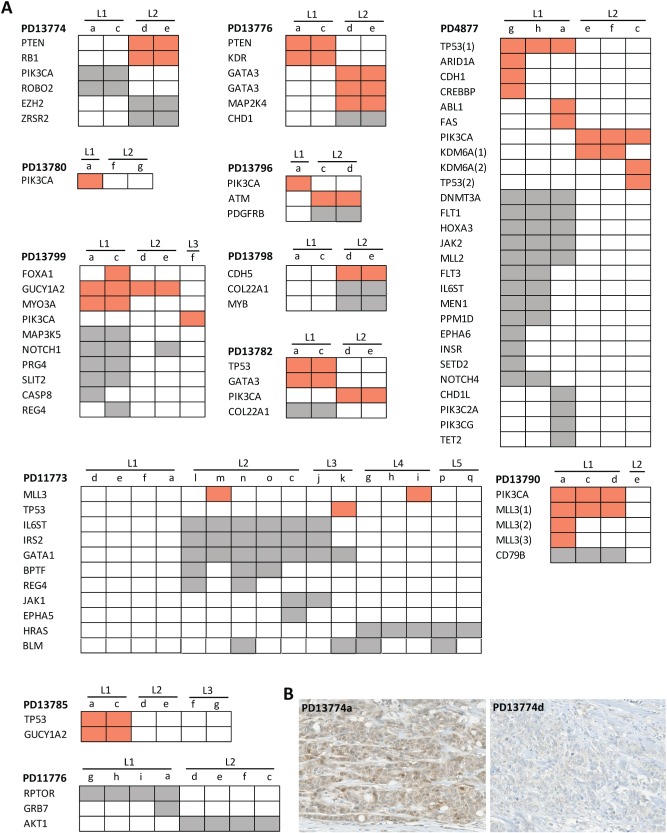
Distribution of non‐silent substitutions and indels in the ‘heterogeneous’ MFBC group. (A) Heat maps representing the non‐silent exonic substitutions and indels of the heterogeneous multifocal lesions, which have no mutation in common between the lesions. The colour code and abbreviations are the same as in Figure 1. (B) Immunohistochemical staining illustrating the loss of PTEN at the protein level only in the lesion of patient PD13774 carrying the PTEN mutation.

By comparing the mutations identified in the various samples and lesions of a single patient, we arbitrarily classified the patients into three groups: those for which all samples from all lesions carried the same mutations (11/36 patients, 31%, Figure 1); those with both common and private mutations (13/36 patients, 36%, Figure 2); and those with no single mutation in common among all samples from the investigated lesions (12/36 patients, 33%, Figure 3). These groups will further be referred to as ‘homogeneous’, ‘intermediate’, and ‘heterogeneous’, respectively. There was no significant association between the number of samples and lesions that were sequenced per patient and the group to which they were categorized (Supplementary Figures 2C and 2D). In contrast, these groups were significantly associated with the total number of mutations per patient: patients from the homogeneous group had fewer mutations than patients from the heterogeneous group (median number of mutations: 2.5 and 4.5, respectively; *p* = 0.030; Supplementary Figure 2E). Nevertheless, the number of mutations *per lesion* between those two groups of multifocal tumors was not significantly different (*p* = 0.568). Of note, the vast majority of the homogeneous and intermediate cancers (9/11 and 9/13) shared at least one oncogenic mutation, potentially representing common and early events during MFBC carcinogenesis.

In the intermediate group, the median allelic frequency of private mutations (ie those not shared by all samples of a given patient) was lower than that of common mutations (0.13 versus 0.26, *p* = 0.042 using a Wilcoxon's signed rank test for paired data). Although copy losses could account for this observation, we believe that the emergence of private, subclonal alterations provides a more general and biologically plausible explanation.

In the heterogeneous group, most cancers were characterized by the presence of different oncogenic mutations in the investigated lesions. In six patients, oncogenic *PIK3CA* mutations were only present in one of the lesions. A similar observation was found for oncogenic *TP53* (*n =* 3), *GATA3* (*n =* 3), and *PTEN* (*n =* 2) mutations. We further demonstrated that the *PTEN* mutation resulted in the loss of PTEN staining by immunohistochemistry only in the lesion carrying the mutation, as exemplified for patient PD13774 in Figure 3B.

Finally, we observed a case of possible convergent evolution for patient PD4877, with each lesion carrying a different *TP53* oncogenic mutation (all three samples from the first lesion carried the R196^*^ mutation, whereas one sample from the other lesion was characterized by the R273H variant).

Altogether, these results suggest that although multifocal tumours may look identical at pathological evaluation, for a substantial number of patients, their lesions differ in terms of oncogenic mutations.

### Association of inter‐lesion heterogeneity with clinico‐pathological variables

We further sought to investigate whether inter‐lesion heterogeneity was associated with clinical or histopathological variables, such as age at diagnosis, axillary lymph node involvement, tumour size, molecular subtype of the tumour based on ER and HER2 status, histological grade, number of multifocal lesions, largest distance between lesions, presence of *in situ* component, and lymphovascular invasion. The only association found was that of inter‐lesion distance: the lesions from patients of the heterogeneous group were further apart from each other than those from patients of the homogeneous group, although the *p* value was of borderline significance (*p* = 0.072). A significant *p* value was, however, reached when comparing patients whose lesions had shared oncogenic mutations with those who had only private oncogenic mutations (*p* = 0.015, Supplementary Figure 3).

### Genome‐wide comparison of multifocal lesions

**Figure 4:**
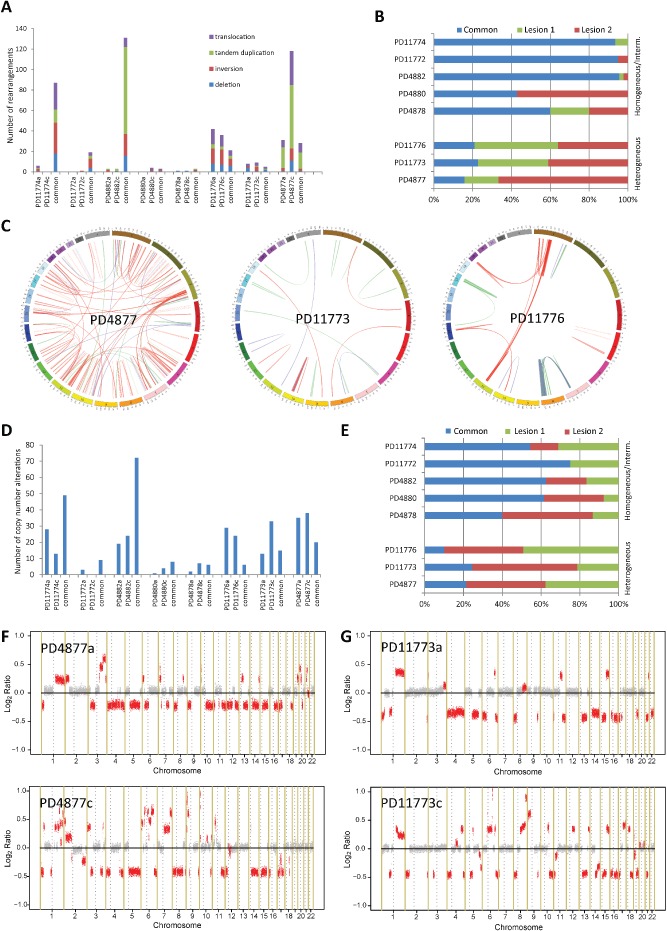
Genome‐wide alterations. Bar plots showing the distribution and the type of somatic rearrangements (A) and the percentage of private and common (present in all investigated lesions from that patient) rearrangements (B). (C) Genome‐wide Circos plots of somatic rearrangements of the three patients classified in the ‘heterogeneous’ group. The chromosomes are represented in the outer ring. Within the inner ring, each blue line represents a common rearrangement, whereas each green or red line represents a rearrangement private to lesion 1 or 2, respectively. Bar plots showing the distribution (D) and the percentage of private and common (present in all investigated lesions from that patient) SCNAs (E). (F, G) Log_2_‐based estimate (log_2_ ratio) of SCNAs, represented in light red, of two of the three patients classified as ‘heterogeneous’.

Targeted mutation screening underestimates the number of common genetic alterations among samples because it only interrogates a tiny fraction of the genome. To investigate the clonal relationship between the lesions, we carried out a low‐coverage screen of somatic structural variation and copy number aberrations (SCNAs) for the eight patients with available frozen material (Figure 4, Supplementary Figure 4, and Supplementary Tables 5–8). Two patients belonged to the homogeneous, three to the intermediate, and three to the heterogeneous group of MFBC tumours, as defined by targeted sequencing. Interestingly, we observed common rearrangements and SCNAs between the lesions for all patients, even for those belonging to the heterogeneous groups, implying that different lesions from the same patient are genetically related. Nevertheless, we cannot exclude that some of the common rearrangements might have arisen during mammary gland development and/or ageing. Although the numbers are too small to draw any statistical conclusion, we further observed a higher proportion of private rearrangements and SCNAs for patients belonging to the heterogeneous group, suggesting an early divergent parallel evolution of the lesions. Of note, most rearrangements did not involve a known cancer gene. Nevertheless, we observed a tandem duplication involving the oncogene *MYC* in the second lesion of patient PD4877 (Supplementary Table 5). At the copy number level, inter‐lesion differences involving cancer‐related genes were present, such as, for example, PTEN loss in only one of the lesions of patients PD4877, PD4878, and PD11773, and MYC amplification in one lesion from PD11776 (Supplementary Tables 7 and 8).

## Discussion

The College of American Pathologists currently considers it sufficient for the immunohistochemical characterization of multifocal lesions with similar grade and histology to be based on the largest lesion 14. Previous studies have demonstrated that despite the similarity of pathological features, lesions might differ in some cases in terms of ER and HER2 receptor status, potentially impacting the treatment and management of these cancers 15, 16, 17. Here we sought to investigate more deeply the intra‐patient, inter‐lesion tumour heterogeneity of MFBCs, and the potential clinical implications. To this end, we used a targeted sequencing approach that specifically focused on cancer‐related genes, similar to those currently used by several molecular cancer profiling initiatives (reviewed in ref 29), on a series of 171 tumour samples from 36 clinically well‐annotated MFBC patients.

Our study showed that 67% (24 patients) of MFBC lesions share all or a fraction of the identified mutations by targeted sequencing. Most of these lesions (18/24) also shared oncogenic variants, suggesting a common origin in the tumour development. Surprisingly, in one‐third of the patients (12 patients), the lesions did not share any mutations despite similar histopathological features. The lesions in all but one patient actually differed in terms of oncogenic mutations. For example, four patients were heterogeneous in terms of oncogenic *TP53* mutations, in line with a recent observation 30. These differences in the presence of oncogenic mutations suggest that different genetic alterations might have been causally involved in the development of the respective lesions in a substantial proportion of MFBCs.

Although the numbers in our study are too small to draw definitive conclusions, we did not observe any significant associations between inter‐lesion heterogeneity and commonly used clinico‐pathological features, with the exception of the inter‐lesion distance. Indeed, MFBCs whose lesions shared oncogenic variants were closer to each other than lesions not sharing any oncogenic variant. This observation supports the concept underlying the historical definition of multicentric and multifocal tumours – namely, that lesions in close proximity to each other are more likely to be biologically similar than lesions that are far apart 1.

The fact that lesions from one‐third of the MFBCs that we studied harboured distinct oncogenic mutations may have substantial therapeutic implications in the context of genotype‐driven trials. Although these trials are mainly running in the metastatic setting, most of them allow identification of the mutation(s) to be performed in the primary tumour 29. In the case of MFBC, the identification of the molecular targets, for example *PIK3CA* and *PTEN*, would differ depending on the lesion interrogated. Our results therefore suggest that ideally all lesions from patients with MFBC should be evaluated, in particular when the lesions are relatively distant from each other.

Another question, especially in the case of heterogeneous MFBCs, is whether MFBC represents clonally related outgrowths of a common ancestral cancer cell or completely separate, coincidental cancers. This has been a matter of debate in the literature during the last few decades 31, 32, 33, 34. Our targeted sequencing data support a common origin in at least two‐thirds of MFBCs. Our genome‐wide analyses, which are limited to a subset of the patients that we studied, suggest that an even greater proportion of MFBCs, including those without any common oncogenic mutations, are clonally related and consequently that the lesions arise through intra‐mammary spread of the tumour cells. The intrinsically invasive nature of the cells making up MFBCs might explain the worse prognostic features associated with multifocal compared with unifocal cancers.

Since the number of molecular alterations with potential clinical utility is rapidly growing in parallel with the increasing number of targeted therapies, our findings suggest that molecularly characterizing only the largest lesion is not sufficient to adequately manage MFBCs, especially when lesions are further apart from each other. The implications of inter‐lesion differences in terms of treatment response to various standard and targeted therapies, as well as in terms of disease progression, deserve further investigation. Recognizing the potential molecular heterogeneity of these cancers has great potential clinical relevance and already represents an important step towards the personalized treatment of this disease.

## Author contributions

CD and CS conceived, designed, and directed the study. CD, GZ, AB, and CS were involved in funding acquisition. DF, EP, DV, MM, NK, IM, LS, DA, DL, MP, and CS provided study materials or patients. CD, DF, EP, FR, SM, MM, DV, NK, RS, RdW, and DL were involved in sample preparation and pathological characterization. CD, SNZ, GZ, and GG analysed and curated substitutions and indels, and generated the oncogenicity calls. GZ, AG, EC, TVB, AB, and DL conducted the resequencing experiments. CD, DF, and GZ analysed the resequencing experiments. CD, SNZ, LM, and SM carried out the whole‐genome rearrangement screen experiments. CD, DF, SNZ, and DB analysed the whole‐genome rearrangement data. BB and DB conducted the copy number analyses. GZ performed the statistical analyses. CD, DF, EP, GZ, DB, SNZ, GG, SL, TVB, DA, DL, MP, DL, PJC, and CS critically interpreted the data and results. CD prepared the manuscript. All authors were involved in reviewing and commenting on the manuscript, and gave their approval of the submitted version of the manuscript.

**SUPPORTING INFORMATION ON THE INTERNET**

The following supporting information may be found in the online version of this article:

**Supplementary methods.**

**Figure S1.** Validation of the mutations using the alternative sequencing platform.

**Figure S2.** Relationship between the mutational burden, the number of samples and lesions interrogated per patient, and the group of MFBCs.

**Figure S3.** Inter‐lesion heterogeneity and inter‐lesion distance.

**Figure S4.** Genome‐wide copy number alterations.

**Table S1.** Patient and tumour's characteristics.

**Table S2.** List of all tumour samples and the applications they have been used for.

**Table S3.** List of genes interrogated by targeted sequencing.

**Table S4.** Somatic substitutions and indels.

**Table S5.** Physical and sequence coverage from the whole‐genome sequencing for the identification of the rearrangements.

**Table S6.** List of validated private and common rearrangements.

**Table S7.** Absolute copy number status of the focal regions reported to be specifically amplified in breast cancer according to ref 28, for the 16 samples with available data.

**Table S8.** Absolute copy number status of the focal regions reported to be specifically deleted in breast cancer according to ref 28, for the 16 samples with available data.

## Supporting information

AppendixS1. SUPPORTING INFORMATION

**FigureS1. Validation of the mutations using the alternative sequencing platform.** (A) Histogram representing the distribution of the coverage of the interrogated mutations across all samples. (B‐C) Scatterplot and Receiver Operating Characteristic (ROC) analyses illustrating the concordance of the allelic frequencies estimated by the two sequencing technologies.

**FigureS2. Relationship between the mutational burden, the number of samples and lesions interrogated per patient, and the group of MFBCs.** (A‐B) Boxplots of mutational burden per patient in terms of the number of lesions and samples that have been interrogated per patient, respectively; (C‐D) Boxplots of the number of interrogated lesions and samples per patient in terms of the group of MFBC, respectively; and (E) Boxplot of the mutational burden per patient in terms of the group of MFBC.

**FigureS3. Inter‐lesion heterogeneity and inter‐lesion distance.** (A) Boxplot of inter‐lesion heterogeneity in terms of oncogenic mutations and largest inter‐lesion distance. Here patients were classified in two groups: those sharing oncogenic mutations between their lesions and those only having oncogenic mutations private to some of their lesions. Patients without identified oncogenic mutations were not considered here. (B) Boxplot of inter‐lesion heterogeneity in terms of the three groups identified according to the targeted sequencing data considering all mutations, and largest inter‐lesion distance.

**FigureS4. Genome‐wide copy number alterations.** Log_2_ based estimate of copy number (Log_2_ Ratio) aberrations, represented in red, across the patients with available whole genome sequencing data that were not represented in Figure 4.

**TableS1.** Patient and tumour's characteristics.

**TableS2.** List of all tumour samples and the applications they have been used for.

**TableS3.** List of genes interrogated by targeted sequencing.

**TableS4.** Somatic substitutions and indels. Abbreviations: WT = wild type; MT = mutated; AF = allelic fraction; NA = not available; sub = substitution; del = deletion; ins = insertion. Note concerning the ‘Validation. IonTorrent’ column: ‘Confirmed’ means that the particular mutation was detected by both technologies in that given sample. ‘Present’ means that the particular mutation was not detected by Illumina, but was detected in the validation phase in that particular sample.

**TableS5.** Physical and sequence coverage from the whole‐genome sequencing for the identification of the rearrangements.

**TableS6.** List of validated private and common rearrangements.

**TableS7.** Absolute copy number status of the focal regions reported to be specifically amplified in breast cancer according to ref 28, for the 16 samples with available copy number data.

**TableS8.** Absolute copy number status of the focal regions reported to be specifically deleted in breast cancer according to ref 28, for the 16 samples with available copy number data.
