# Supplementary material for: Uncovering the genomic heterogeneity of multifocal breast cancer
Source: J Pathol. 2015 May 7;236(4):457–66. doi: 10.1002/path.4540 (PMC4691324; doi:10.1002/path.4540)
Supplement: Supplementary file 1 — AppendixS1. SUPPORTING INFORMATION [file path0236-0457-sd1.docx]

**SUPPORTING INFORMATION**

**Supplementary methods**

**Characterization of the samples**

The ER status was obtained using the anti-estrogen receptor antibody [SP1] (ab166600, Abcam®, Cambridge, UK). The staining was scored accordingly to Allred [35,36] using a combined score for proportion and intensity, and was considered as positive if the global score was >2. The HER2 status was defined using the antiHER2/neu antibody (4B5) (Roche). The scoring and subsequent FISH-analyses were done in accordance to the ASCO-CAP Guidelines on HER2-testing [37]. The histological grade was defined using the modified Bloom-Richardson grading system [38,39]. Ki67 was defined using the Monoclonal Mouse Anti-Human Ki-67 Antigen (Clone MIB-1) (Dako, Glostrup, Denmark). The PTEN status was investigated by immunohistochemistry using the SP218 clone from Diagomics. Histological grade and type, as well as ER and HER2 status were centrally reviewed. The percentage of tumor epithelial cells was also evaluated prior to DNA extraction. Only samples with a cellularity of >40% were considered. In case the cellularity was below 40%, the sample was only considered if macrodissection was feasible to enrich for tumor cells. For the negative lymph nodes serving as source of germline DNA (Table S1), two hematoxylin and eosin slides, one before and one after extraction, were carefully examined by a pathologist to exclude the presence of invasive epithelial cells.

The inter-lesion distance was assessed by the pathologists on the surgical specimen as the in situ and invasive tumor-free distance between 2 invasive lesions. This information was not available for 14 patients, but could be retrieved for 4 patients from the medical images (PD13780, PD13782, PD13790 and PD13800).

**DNA and RNA extraction protocols**

Genomic DNA from the clinical frozen samples and from the FFPE samples was extracted using the Qiagen DNeasy Blood & Tissue Kit and Qiagen QIAamp DNA FFPE Tissue kit respectively, according to the supplier’s instructions (Qiagen, Hilden, Germany). Both kits included a proteinase K digestion at 55°C overnight. DNA quantity was defined using the QUBIT 2.0 Fluorometer (Invitrogen).

**Targeted gene screen experiments**

Five samples (PD4878d, PD4878e, PD4880d, PD13804a and PD13804e) could not be sequenced for the initial targeted sequencing with Illumina due to limited DNA quantity. These samples were however interrogated in the validation phase for the mutations which were identified in the other samples from the same patient.

Prior to sequencing, samples were subjected to a Sequenom experiment of 96 SNPs. The sequenced SNPs were then compared to the Sequenom SNPs for the same sample. This was used to verify that no sample mismatch occurred during the sequencing experiment. During the data analysis part, we further genotyped all samples within the study against each other using common sequenced SNPs, to independently confirm which samples belonged to the same patient.

**Mutation revalidation procedures**

Target amplicons, on average 150 base pairs (bp) (range 125 -175 bp) long, were designed with the Ion AmpliSeq™ Designer system ([www.ampliseq.com](http://www.ampliseq.com)) in two batches divided into two test tubes each. All reactions were carried out in the conditions described by the manufacturer. Ten ng from each sample were amplified using the Ion AmpliSeq™ Library Kit 2.0 (Life Technologies Inc.). Before the partial digestion with FuPa Reagent, a unique barcode per DNA sample was added to each sample library with the Ion Xpress™ Barcode Adapters (Life Technologies). Libraries were then purified with Agencourt® AMPure® XP Reagent (Beckman Coulter) magnetic system and quantified with an Agilent 2100 Bioanalyzer system. Libraries (range 9-16) were finally pooled at a final 50 pmol/l concentration. Then, we performed emulsion PCR with the Ion OneTouch™ 200 Template Kit v2 DL (Life Technologies) in the Ion OneTouch™ System (Life Technologies) to generate template-positive Ion Sphere™ Particles (ISPs) and, after that, we enriched them with magnetic Dynabeads® MyOne™ Streptavidin C1 (Invitrogen®, Life Technologies Inc.) by using Ion OneTouch™ Enrichment System (ES), in the conditions described by the manufacturer. Template-positive ISPs were mixed with 5 μl Control ISPs (Life Technologies) in 100 μl of Annealing Buffer (Life Technologies), centrifuged, resuspended in 15 μl of Annealing Buffer (Life Technologies) and mixed with 12 μl Sequencing Primer. Three microlitres of Ion Torrent Personal Genome Machine® (PGM™) 200 Sequencing Polymerase was added to the mixture, and the whole reaction volume of about 30 μl was loaded onto Ion 318™ Chips V2(Life Technologies, Inc.). Sequencing was carried out with the Ion Torrent PGM™ 200 Sequencing Kit (Life Technologies) in the Ion Torrent PGM™ System (Life Technologies). Alignment and calling were performed using the Ion Torrent Server Suite, with default parameters for FFPE analysis.

**Calling of substitutions and indels**

To enrich for high-confidence somatic variants that impact on protein function further filtering was conducted using the following criteria: 1/ removal of variants present within regions prone to sequence context specific artifacts, including regions of high depth and low mapping quality; 2/ removal of all single base insertions or deletions adjacent to regions of more than 5 homopolymer bases (e.g. insertion of A immediately next to AAAAA); and 3/ removal of highly recurrent calls that presented with a narrow allele distribution.

**Rearrangement screen experiments and analysis**

The mean target genome-wide sequence physical coverage was 12.6. Short reads were mapped back to the reference genome (GRCh37) and discordantly mapping reads were identified as pairs that did not map with the expected insert size, mapped in the wrong orientation or mapped to different chromosomes, as previously described [23,40]. At least three reads were required to report a putative rearrangement in the discovery screen. For putative rearrangements, primers mapping on either end of the reported rearrangement were designed and amplified by conventional PCR in tumor DNA, and blood-derived normal DNA. PCR reactions were performed in duplicate and amplimers were separated by agarose gel electrophoresis. Conventional Sanger sequence of amplimers specific to tumor samples enabled breakpoint resolution to base pair level. Sanger sequences were mapped to the reference genome and genomic breakpoints were defined.

Rearrangement classification was in accordance with Stephens *et al* [23]. Briefly, somatic rearrangements were classified as deletions if reads mapped at distances less than expected for insert size and were associated with a reduction in copy number for deletions exceeding 10kb, tandem duplications if reads mapped at increased distances, had the correct orientation and an associated copy number increased for variants exceeding 10kb, inversions if reads mapped in inappropriate orientation or inter-chromosomal if discordantly mapping reads mapped to different chromosomes. Features at breakpoint junctions were also delineated including microhomology and non-templated sequence.

The rearrangements were annotated with the fusion flag as in previous publications [40]. In brief, the higher the number, the more “interesting” the fusion could be. The definition of the codes is listed below.

- 910: In frame fusion gene, 2 different genes, broken in coding exons. Same orientation. Reading frame preserved
- 900: In frame fusion gene, 2 different genes, broken in coding introns. Same orientation. Reading frame preserved
- 890: In frame fusion gene, same gene, broken in 2 different coding exons. Same orientation. Reading frame preserved
- 880: In frame fusion gene, same gene, broken in 2 different coding introns. Same orientation. Reading frame preserved
- 860: 5UTR to 5UTR fusion, different genes. Same orientation
- 840: 5UTR to 5UTR fusion, same gene. Same orientation
- 820: 5UTR to coding fusion, 2 different genes. 1 break in 5UTR, 1 break in coding region. Same orientation. Ambiguous reading frame
- 800: 5UTR to coding fusion, same gene. 1 break in 5UTR, 1 break in coding region. Same orientation. Ambiguous reading frame
- 780: Fusion, 2 different genes, broken in coding exons. Same orientation. Ambiguous reading frame
- 760: Fusion, same gene, broken in coding exons. Same orientation. Ambiguous reading frame
- 740: Fusion, 2 different genes, broken in ambiguous coding region. Same orientation. Ambiguous reading frame
- 720: Fusion, same gene, broken in ambiguous coding region. Same orientation. Ambiguous reading frame
- 715: Truncated protein product. Stop Codon formed at breakpoint junction
- 710: Out of frame fusion gene, 2 different genes, broken in coding exons. Same orientation. Reading frame different
- 700: Out of frame fusion gene, 2 different genes, broken in coding introns. Same orientation. Reading frame different
- 690: Out of frame fusion gene, same gene, broken in coding exons. Same orientation. Reading frame different
- 680: Out of frame fusion gene, same gene, broken in 2 different coding introns. Same orientation. Reading frame different
- 660: 5UTR to 3UTR fusion, different genes. Same orientation
- 640: 5UTR to 3UTR fusion, same gene. Same orientation
- 620: 3UTR to coding fusion, 2 different genes. 1 break in 3UTR, 1 break in coding region. Same orientation. Ambiguous reading frame
- 600: 3UTR to coding fusion, same gene. 1 break in 3UTR, 1 break in coding region. Same orientation. Ambiguous reading frame
- 580: fusion, 2 different genes. 1 break in 5UTR, 1 break in coding region. Opposite orientation. Ambiguous reading frame
- 570: fusion, same gene. 1 break in 5UTR, 1 break in coding region. Opposite orientation. Ambiguous reading frame
- 560: fusion, 2 different genes. 1 break in 3UTR, 1 break in coding region. Opposite orientation. Ambiguous reading frame
- 550: fusion, same gene. 1 break in 3UTR, 1 break in coding region. Opposite orientation. Ambiguous reading frame
- 540: fusion, 2 different genes, broken in coding introns. Opposite orientation. Ambiguous reading frame
- 530: fusion, same gene, broken in coding introns. Opposite orientation. Ambiguous reading frame
- 520: Fusion, 2 different genes, broken in coding exons. Opposite orientation. Ambiguous reading frame
- 510: Fusion, same gene, broken in coding exons. Opposite orientation. Ambiguous reading frame
- 500: Fusion, 2 different genes, broken in ambiguous coding region. Opposite orientation. Ambiguous reading frame
- 490: Fusion, same gene, broken in ambiguous coding region. Opposite orientation. Ambiguous reading frame
- 460: In frame fusion gene, same gene, broken in same coding intron. Same orientation. Reading frame unchanged
- 450: Fusion, same gene, broken in same coding intron. Different orientation. Ambiguous reading frame
- 440: 3UTR to 5UTR fusion, different genes. Same orientation
- 420: 3UTR to 5UTR fusion, same gene. Same orientation
- 400: 3UTR to 3UTR fusion, different genes. Same orientation
- 380: 3UTR to 3UTR fusion, same gene. Same orientation
- 360: 5UTR to 5UTR fusion, different genes. Different orientation
- 350: 5UTR to 5UTR fusion, same gene. Different orientation
- 320: 5UTR to 3UTR fusion, different genes. Different orientation
- 310: 5UTR to 3UTR fusion, same gene. Different orientation
- 280: 3UTR to 5UTR fusion, different genes. Different orientation
- 270: 3UTR to 5UTR fusion, same gene. Different orientation
- 240: 3UTR to 3UTR fusion, different genes. Different orientation
- 230: 3UTR to 3UTR fusion, same gene. Different orientation
- 200: Intron to something else fusion
- 180: Something else to intron fusion
- 100: Something else to something else fusion
- 0: No fusion
- −1: unknown fusion

**References**

**(Note: reference numbers correspond to reference list in main article)**

23. Stephens PJ, McBride DJ, Lin ML, *et al*. Complex landscapes of somatic rearrangement in human breast cancer genomes. *Nature* 2009; **462:** 1005–1010.

28. Zack TI, Schumacher SE, Carter SL, *et al*. Pan-cancer patterns of somatic copy number alteration. *Nature Genet* 2013; **45:** 1134–1140.

35. Harvey JM, Clark GM, Osborne CK, *et al*. Estrogen receptor status by immunohistochemistry is superior to the ligand-binding assay for predicting response to adjuvant endocrine therapy in breast cancer. *J Clin Oncol* 1999; **17:** 1474–1481.

36. Leake R, Barnes D, Pinder S, *et al*. Immunohistochemical detection of steroid receptors in breast cancer: a working protocol. UK Receptor Group, UK NEQAS, The Scottish Breast Cancer Pathology Group, and The Receptor and Biomarker Study Group of the EORTC. *J Clin Pathol* 2000; **53:** 634–635.

37. Wolff AC, Hammond ME, Schwartz JN, *et al*. American Society of Clinical Oncology/College of American Pathologists guideline recommendations for human epidermal growth factor receptor 2 testing in breast cancer. *J Clin Oncol* 2007; **25:** 118–145.

38. Elston CW, Ellis IO. Pathological prognostic factors in breast cancer. I. The value of histological grade in breast cancer: experience from a large study with long-term follow-up. *Histopathology* 1991; **19:** 403–410.

39. Genestie C, Zafrani B, Asselain B, *et al*. Comparison of the prognostic value of Scarff–Bloom–Richardson and Nottingham histological grades in a series of 825 cases of breast cancer: major importance of the mitotic count as a component of both grading systems. *Anticancer Res* 1998; **18:** 571–576.

40. Nik-Zainal S, Van Loo P, Wedge DC, *et al*. The life history of 21 breast cancers. *Cell* 2012; **149:** 994–1007.

**Supplementary figure and table legends**

**Figure S1. Validation of the mutations using the alternative sequencing platform.** (A) Histogram representing the distribution of the coverage of the interrogated mutations across all samples. (B-C) Scatterplot and Receiver Operating Characteristic (ROC) analyses illustrating the concordance of the allelic frequencies estimated by the two sequencing technologies.

**Figure S2. Relationship between the mutational burden, the number of samples and lesions interrogated per patient, and the group of MFBCs.** (A-B) Boxplots of mutational burden per patient in terms of the number of lesions and samples that have been interrogated per patient, respectively; (C-D) Boxplots of the number of interrogated lesions and samples per patient in terms of the group of MFBC, respectively; and (E) Boxplot of the mutational burden per patient in terms of the group of MFBC.

**Figure S3. Inter-lesion heterogeneity and inter-lesion distance.** (A) Boxplot of inter-lesion heterogeneity in terms of oncogenic mutations and largest inter-lesion distance. Here patients were classified in two groups: those sharing oncogenic mutations between their lesions and those only having oncogenic mutations private to some of their lesions. Patients without identified oncogenic mutations were not considered here. (B) Boxplot of inter-lesion heterogeneity in terms of the three groups identified according to the targeted sequencing data considering all mutations, and largest inter-lesion distance.

**Figure S4. Genome-wide copy number alterations.** Log_2_ based estimate of copy number (Log_2_ Ratio) aberrations, represented in red, across the patients with available whole genome sequencing data that were not represented in Figure 4.

**Table S1.** Patient and tumour’s characteristics.

**Table S2.** List of all tumour samples and the applications they have been used for.

**Table S3.** List of genes interrogated by targeted sequencing.

**Table S4.** Somatic substitutions and indels. Abbreviations: WT = wild type; MT = mutated; AF = allelic fraction; NA = not available; sub = substitution; del = deletion; ins =insertion. Note concerning the ‘Validation.IonTorrent’ column: ‘Confirmed’ means that the particular mutation was detected by both technologies in that given sample. ‘Present’ means that the particular mutation was not detected by Illumina, but was detected in the validation phase in that particular sample.

**Table S5.** Physical and sequence coverage from the whole-genome sequencing for the identification of the rearrangements.

**Table S6.** List of validated private and common rearrangements**.**

**Table S7.** Absolute copy number status of the focal regions reported to be specifically amplified in breast cancer according to ref 28, for the 16 samples with available copy number data.

**Table S8.** Absolute copy number status of the focal regions reported to be specifically deleted in breast cancer according to ref 28, for the 16 samples with available copy number data.
