# Supplementary figures and images for: Uncovering the genomic heterogeneity of multifocal breast cancer
Source: J Pathol. 2015 May 7;236(4):457–66. doi: 10.1002/path.4540 (PMC4691324; doi:10.1002/path.4540)

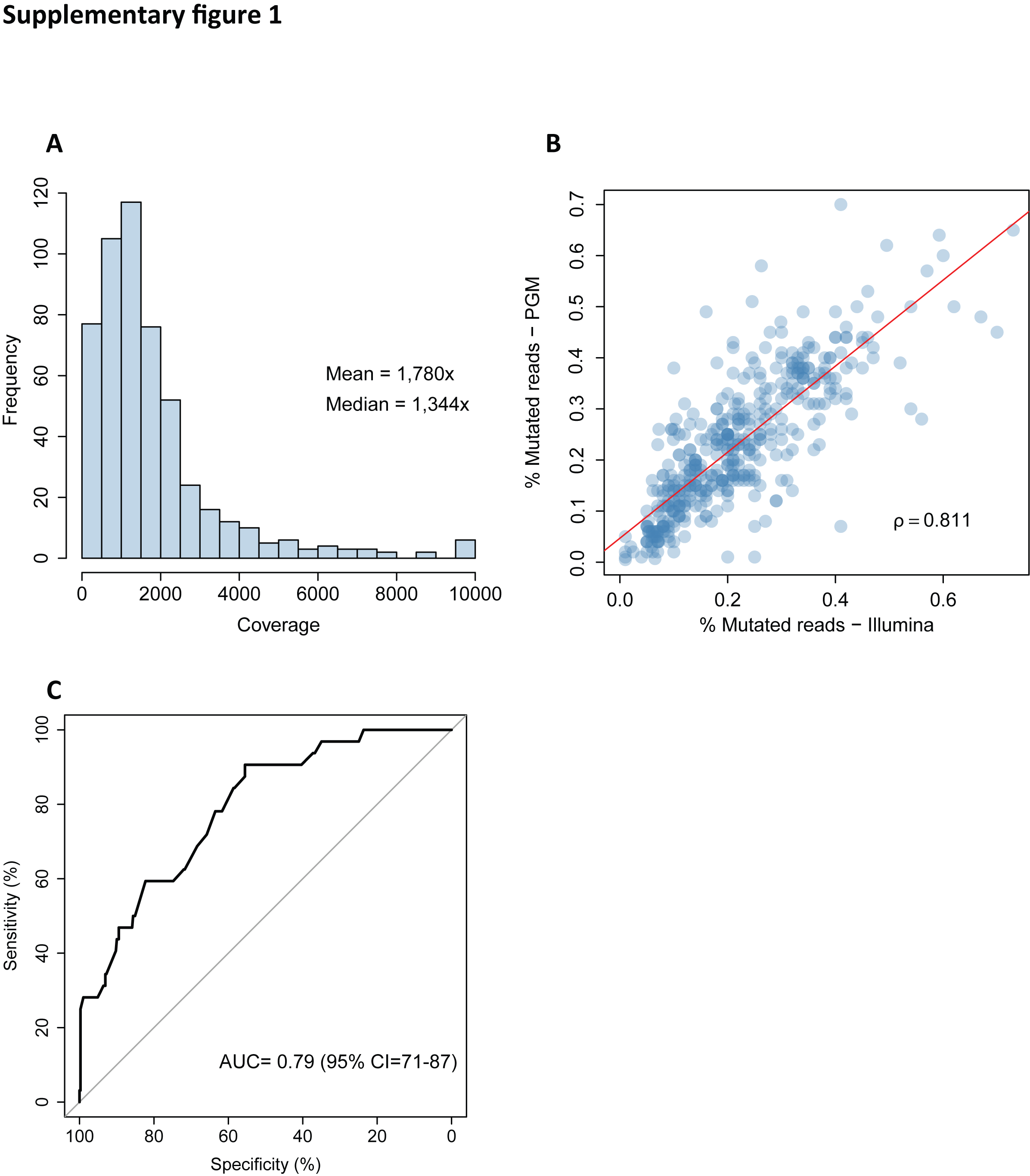

Supplement: Supplementary file 2 — FigureS1. Validation of the mutations using the alternative sequencing platform. (A) Histogram representing the distribution of the coverage of the interrogated mutations across all samples. (B‐C) Scatterplot and Receiver Operating Characteristic (ROC) analyses illustrating the concordance of the allelic frequencies estimated by the two sequencing technologies. [file path0236-0457-sd2.tif]

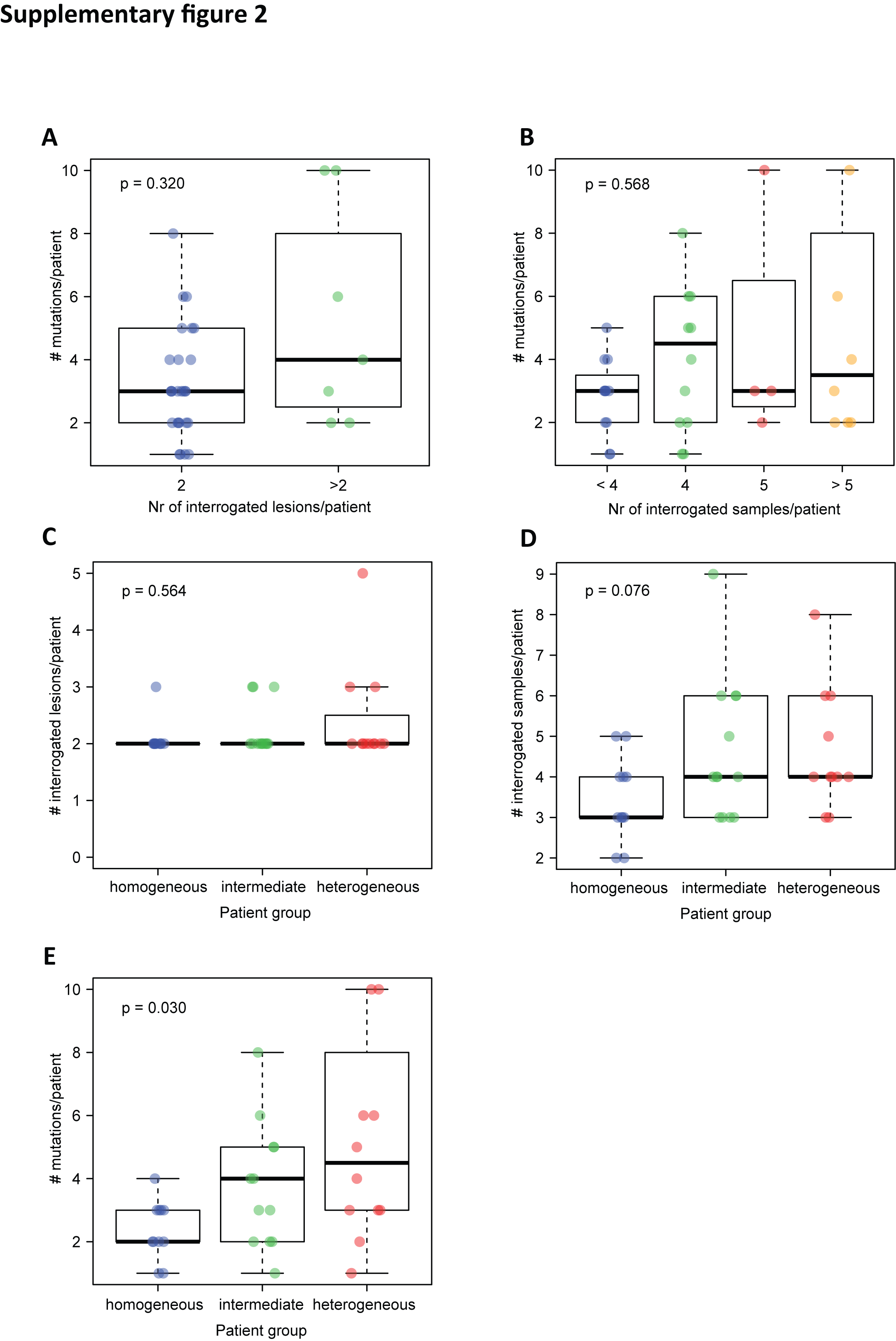

Supplement: Supplementary file 3 — FigureS2. Relationship between the mutational burden, the number of samples and lesions interrogated per patient, and the group of MFBCs. (A‐B) Boxplots of mutational burden per patient in terms of the number of lesions and samples that have been interrogated per patient, respectively; (C‐D) Boxplots of the number of interrogated lesions and samples per patient in terms of the group of MFBC, respectively; and (E) Boxplot of the mutational burden per patient in terms of the group of MFBC. [file path0236-0457-sd3.tif]

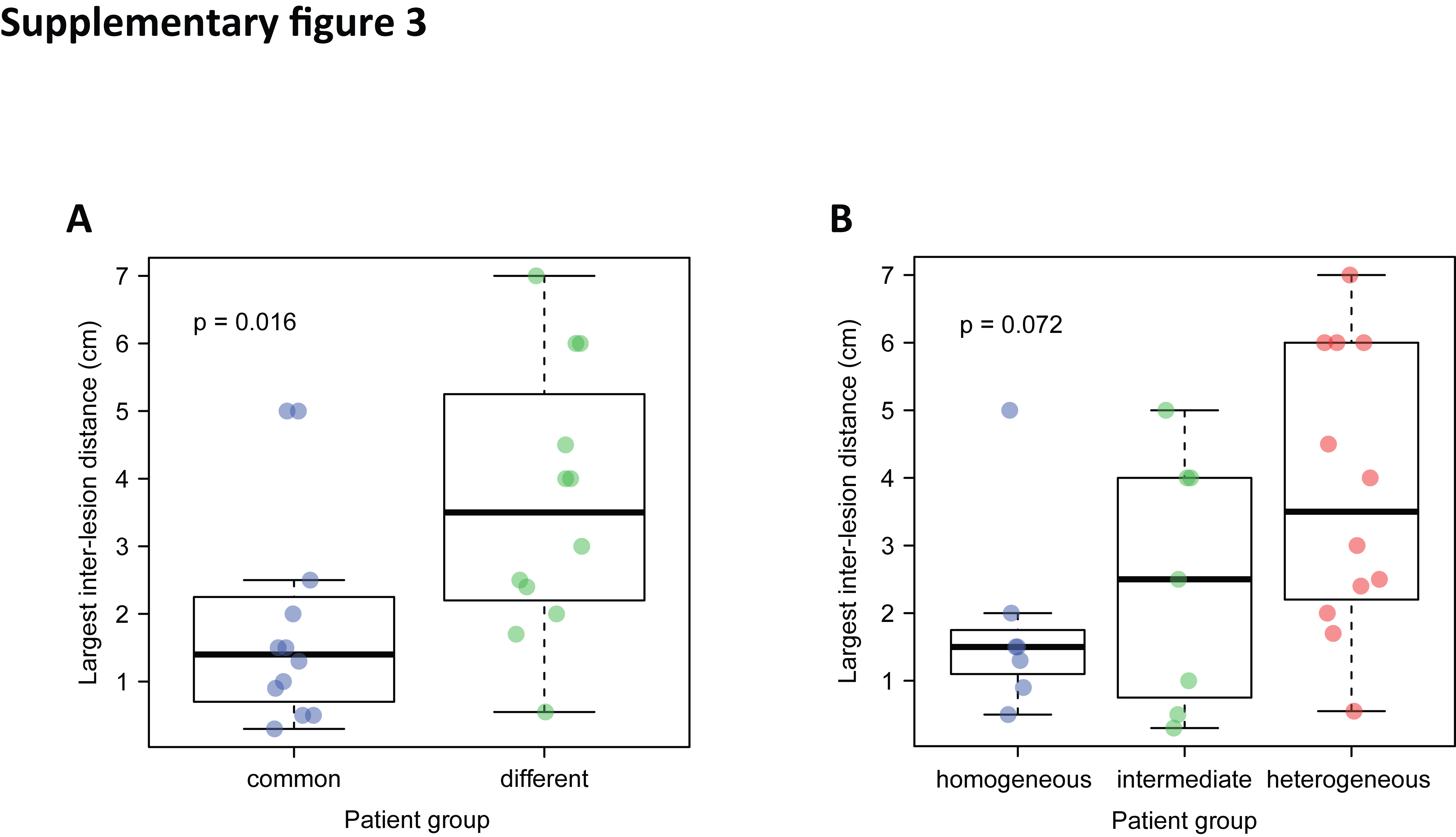

Supplement: Supplementary file 4 — FigureS3. Inter‐lesion heterogeneity and inter‐lesion distance. (A) Boxplot of inter‐lesion heterogeneity in terms of oncogenic mutations and largest inter‐lesion distance. Here patients were classified in two groups: those sharing oncogenic mutations between their lesions and those only having oncogenic mutations private to some of their lesions. Patients without identified oncogenic mutations were not considered here. (B) Boxplot of inter‐lesion heterogeneity in terms of the three groups identified according to the targeted sequencing data considering all mutations, and largest inter‐lesion distance. [file path0236-0457-sd4.tif]

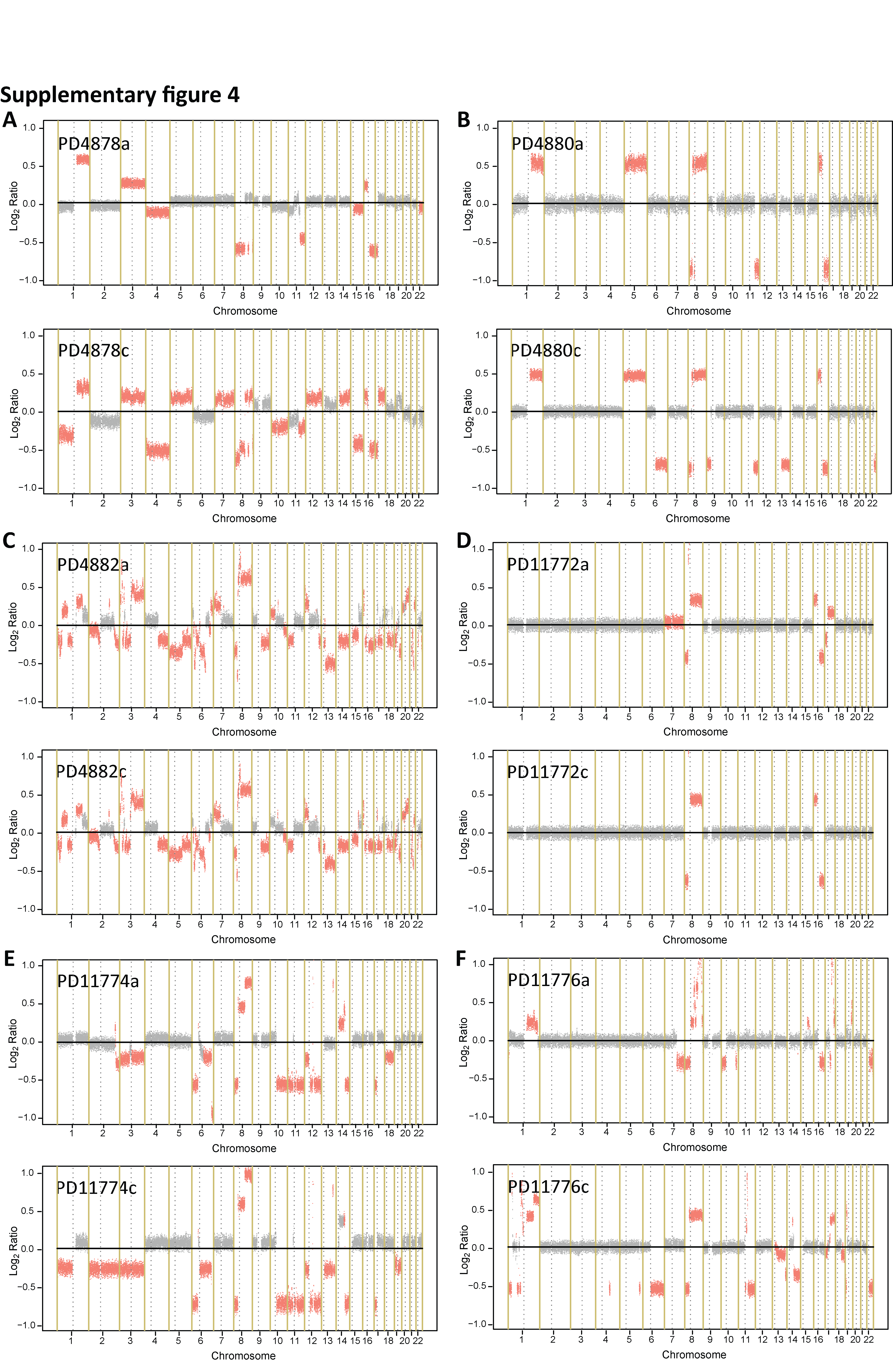

Supplement: Supplementary file 5 — FigureS4. Genome‐wide copy number alterations. Log2 based estimate of copy number (Log2 Ratio) aberrations, represented in red, across the patients with available whole genome sequencing data that were not represented in Figure 4. [file path0236-0457-sd5.tif]
